# The prevalence and characteristics of metabolic syndrome according to different definitions in China: a nationwide cross-sectional study, 2012–2015

**DOI:** 10.1186/s12889-022-14263-w

**Published:** 2022-10-07

**Authors:** Yilin Huang, Linfeng Zhang, Zengwu Wang, Xin Wang, Zuo Chen, Lan Shao, Ye Tian, Congying Zheng, Lu Chen, Haoqi Zhou, Xue Cao, Yixin Tian, Runlin Gao, for the China Hypertension Survey investigators, Liqun Hu, Hongqi Li, Qi Zhang, Guang Yan, Fangfang Zhu, Xianghua Fang, Chunxiu Wang, Shaochen Guan, Xiaoguang Wu, Hongjun Liu, Chengbei Hou, Han Lei, Wei Huang, Nan Zhang, Ge Li, Lihong Mu, Xiaojun Tang, Ying Han, Huajun Wang, Dongjie Lin, Liangdi Xie, Daixi Lin, Jing Yu, Xiaowei Zhang, Wei Liang, Heng Yu, Qiongying Wang, Lan Yang, Yingqing Feng, Yuqing Huang, Peixi Wang, Jiaji Wang, Harry HX Wang, Songtao Tang, Tangwei Liu, Rongjie Huang, Zhiyuan Jiang, Haichan Qin, Guoqin Liu, Zhijun Liu, Wenbo Rao, Zhen Chen, Yalin Chu, Fang Wu, Haitao Li, Jianlin Ma, Tao Chen, Ming Wu, Jixin Sun, Yajing Cao, Yuhuan Liu, Zhikun Zhang, Yanmei Liu, Dejin Dong, Guangrong Li, Hong Guo, Lihang Dong, Haiyu Zhang, Fengyu Sun, Xingbo Gu, Kaijuan Wang, Chunhua Song, Peng Wang, Hua Ye, Wei Nie, Shuying Liang, Congxin Huang, Fang Chen, Yan Zhang, Heng Zhou, Jing Xie, Jianfang Liu, Hong Yuan, Chengxian Guo, Yuelong Huang, Biyun Chen, Xingsheng Zhao, Wenshuai He, Xia Wen, Yanan Lu, Xiangqing Kong, Ming Gui, Wenhua Xu, Yan Lu, Jun Huang, Min Pan, Jinyi Zhou, Ming Wu, Xiaoshu Cheng, Huihui Bao, Xiao Huang, Kui Hong, Juxiang Li, Ping Li, Bin Liu, Junduo Wu, Longbo Li, Yunpeng Yu, Yihang Liu, Chao Qi, Jun Na, Li Liu, Yanxia Li, Guowei Pan, Degang Dong, Peng Qu, Jinbao Ma, Juan Hu, Fu Zhao, Jianning Yue, Minru Zhou, Zhihua Xu, Xiaoping Li, Qiongyue Sha, Fuchang Ma, Qiuhong Chen, Huiping Bian, Jianjun Mu, Tongshuai Guo, Keyu Ren, Chao Chu, Zhendong Liu, Hua Zhang, Yutao Diao, Shangwen Sun, Yingxin Zhao, Junbo Ge, Jingmin Zhou, Xuejuan Jin, Jun Zhou, Bao Li, Lijun Zhu, Yuean Zhang, Gang Wang, Zhihan Hao, Li Cai, Zhou Liu, Zhengping Yong, Shaoping Wan, Zhenshan Jiao, Yuqiang Fan, Hui Gao, Wei Wang, Qingkui Li, Xiaomei Zhou, Yundai Chen, Bin Feng, Qinglei Zhu, Sansan Zhou, Nanfang Li, Ling Zhou, Delian Zhang, Jing Hong, Tao Guo, Min Zhang, Yize Xiao, Xuefeng Guang, Xinhua Tang, Jing Yan, Xiaoling Xu, Li Yang, Aimin Jiang, Wei Yu

**Affiliations:** 1 Division of Prevention and Community Health, National Center for Cardiovascular Disease, National Clinical Research Center of Cardiovascular Diseases, State Key Laboratory of Cardiovascular Disease, Fuwai Hospital, Peking Union Medical College & Chinese Academy of Medical Sciences, No. 15 (Lin), Fengcunxili, Mentougou District, Beijing, 102308 China; 2 Department of Cardiology, National Center for Cardiovascular Disease, National Clinical Research Center of Cardiovascular Disease, State Key Laboratory of Cardiovascular Disease, Fuwai Hospital, Peking Union Medical College & Chinese Academy of Medical Sciences, No. 167, Beilishilu, Xicheng District, Beijing, 100037 China

**Keywords:** Metabolic syndrome, Prevalence, China

## Abstract

**Background:**

Metabolic syndrome (MetS) is characterized by a cluster of signs of metabolic disturbance and has caused a huge burden on the health system. The study aims to explore the prevalence and characteristics of MetS defined by different criteria in the Chinese population.

**Methods:**

Using the data of the China Hypertension Survey (CHS), a nationally representative cross-sectional study from October 2012 to December 2015, a total of 28,717 participants aged 35 years and above were included in the analysis. The MetS definitions of the International Diabetes Federation (IDF), the updated US National Cholesterol Education Program Adult Treatment Panel III (the revised ATP III), and the Joint Committee for Developing Chinese Guidelines (JCDCG) on Prevention and Treatment of Dyslipidemia in Adults were used. Multivariable logistic regression was used to identify factors associated with MetS.

**Results:**

The prevalence of MetS diagnosed according to the definitions of IDF, the revised ATP III, and JCCDS was 26.4%, 32.3%, and 21.5%, respectively. The MetS prevalence in men was lower than in women by IDF definition (22.2% vs. 30.3%) and by the revised ATP III definition (29.2% vs. 35.4%), but the opposite was true by JCDCG (24.4%vs 18.5%) definition. The consistency between the three definitions for men and the revised ATP III definition and IDF definition for women was relatively good, with kappa values ranging from 0.77 to 0.89, but the consistency between the JCDCG definition and IDF definition (kappa = 0.58) and revised ATP III definition (kappa = 0.58) was poor. Multivariable logistic regression showed that although the impact and correlation intensity varied with gender and definition, area, age, education, smoking, alcohol use, and family history of cardiovascular disease were factors related to MetS.

**Conclusions:**

The prevalence and characteristics of the MetS vary with the definition used in the Chinese population. The three MetS definitions are more consistent in men but relatively poor in women. On the other hand, even if estimated according to the definition of the lowest prevalence, MetS is common in China.

**Supplementary Information:**

The online version contains supplementary material available at 10.1186/s12889-022-14263-w.

## Background

MetS is a syndrome clustering, including fat metabolism disorder, obesity, diabetes, insulin resistance, and other risk factors, increasing cardiovascular diseases (CVDs) [1]. Convincing evidence shows that metabolic syndrome (MetS) has been a growing public health problem worldwide. The prevalence of MetS is high and is expected to continue rising in developed and developing countries [2–4]. Exploring the characteristics and prevalence of metabolic syndrome may provide important public health implications for preventing and managing CVDs.

In the past few decades, several international organizations had provided the definitions of MetS. The World Health Organization (WHO) 1998 first attempted to put forward a diagnostic criterion of metabolic syndrome [5] the US National Cholesterol Education Program Adult Treatment Panel III (NCEP-ATP III) proposed diagnostic criteria of 5 components in 2001 to facilitate clinical diagnosis of high-risk individuals [6], the American Heart Association/National Heart, Lung, and Blood Institute updated the ATP III definition in 2005 (the revised ATP III) [7], and International Diabetes Federation (IDF) recommended a new definition in 2006 [8]. In China, the Joint Committee for Developing Chinese Guidelines (JCDCG) on Prevention and Treatment of Dyslipidemia in Adults suggested a Chinese definition for MetS in 2016 [9].

Depending on the definition used, estimates of the prevalence of MetS vary worldwide [10–12], and there is a clear difference. In recent studies, the MetS was prevalent in 24.6% of men and 23.8% of women in China according to ATP III criteria [13], 21.8% of men and 45.6% of women in Iran in 2021 according to IDF definition [14], 32.8% of men and 36.6% women according to ATP III criteria in 2011–2012 in the United States [15]. Using various criteria, the prevalence in China ranged from 9.82% to 48.8% [13, 16, 17], which led to confusion and a lack of comparability among studies. Therefore, it is necessary to report and compare the prevalence of MetS by different criteria, which may be helpful for researchers to understand MetS better and formulate a more scientific definition.

Although many epidemiological studies on MetS were conducted on the Chinese population in recent years, there is little national information on the prevalence of different MetS definitions. In the WHO definition, insulin resistance is regarded as a prerequisite, which limits its use [5]. Therefore, in this study, we will use the data of the China Hypertension Survey (CHS) to explore the prevalence and characteristics of MetS according to IDF, the revised ATP III, and JCDCG criteria.

## Methods

### Design and study population

The CHS was a cross-sectional study conducted between October 2012 and December 2015, and the study design was published previously [18, 19]. Briefly, A nationally representative sample of the general Chinese population across all 31 provinces in mainland China was obtained using a stratified multistage random sampling method. In this sub-study, 262 sampled urban cities and rural counties in the CHS were stratified into eastern, central, and western regions according to geographical location and economic level, and 16 cities and 17 counties were selected with a simple random sampling method, including 7 cities and 7 counties from the eastern regions, 6 cities and 6 counties from the central regions, and 3 cities and 4 counties from the western regions. Then, at least three communities or villages were randomly selected from each city or county. To meet the designed sample size of 35,000 participants aged ≥ 35 years and take nonresponses into account, 56,000 subjects were randomly selected from the eligible sites. Finally, 34,994 participants completed the survey, with an overall response rate of 62.5%. After excluding the pregnant or lactating (*n* = 163) women and the subjects with incomplete demographic data (*n* = 925) and laboratory tests(*n* = 5189), 28,717 subjects aged ≥ 35 years were included in the final analysis. The comparison of the characteristics of the subjects participating in the study and those not participating in the analysis can be found in Appendix Table 1.

Written informed consent was obtained from each participant. The Ethics Committee of Fuwai Hospital (Beijing, China) approved this study.

### Data collection

All study investigators and staff members were trained according to the study protocol. A standardized questionnaire developed by the coordinating center, Fuwai Hospital, was administered to obtain information on demographic characteristics factors, such as age, area, education level, smoking status and alcohol use, and family history of cardiovascular disease (CVD). Smoking status was defined as participants who had smoked at least 20 packs of cigarettes in their lifetime and currently smoked cigarettes. Alcohol use was defined as consuming at least one alcoholic beverage per week in the past month. Family history of cardiovascular disease (CVD) referred to that at least one of the parents and siblings had a history of hypertension, dyslipidemia, diabetes, coronary heart disease, or stroke.

Anthropometry data (weight, height, and waist circumference) and blood pressure were measured at the local medical centers. Fasting blood samples were collected in the morning after 10-12 h fasting and were processed properly and refrigerated immediately. Serum glucose, triglycerides (TG) and high-density lipoprotein cholesterol (HDL-C) were determined by automatic biochemical analyzer (Beckman Coulter AU 680). The serum glucose was measured by the hexokinase method, serum TG by GPO-POD method, and HDL-C by automated homogeneous direct measurement method. All samples were analyzed in the central laboratory. Body mass index (BMI) was classified according to the recommendations of Working Group of Obesity in China, < 18.5 kg/m^2^ (underweight), 18.5–23.9 kg/m^2^ (normal range), 24–27.9 kg/m^2^ (overweight), ≥ 28 kg/m^2^ (obesity) [20].

### Diagnosing standard

According to the IDF definition, MetS was defined as central obesity (WC ≥ 90 cm for Chinese men and ≥ 80 cm for Chinese women) along with two or more of the following abnormalities: (1) Elevated triglyceride (TG) > 1.7 mmol/L or receipt of specific treatment for this lipid abnormality; (2) High-density lipoproteins cholesterol (HDL-C) level of 1.03 mmol/L in men and 1.29 mmol/L in women or receipt of specific treatment for this lipid abnormality; (3) Systolic blood pressure ≥ 130 mmHg or diastolic blood pressure ≥ 85 mmHg or receipt of treatment of previously diagnosed hypertension; (4) Fasting plasma glucose (FPG) level of 5.6 mmol/L or previously diagnosed type 2 diabetes [8].

According to the revised ATP III definition, MetS was defined as if there were more than three or more of the following abnormalities: (1) Central obesity (WC $\ge$ 90 cm for men and $\ge$ 80 cm for women); (2) Elevated triglyceride level $\ge$ 1.7 mmol/L or on drug treatment for elevated triglycerides; (3) Reduced HDL-C < 40 mg/dL (1.03 mmol/L) in men; < 50 mg/dL (1.3 mmol/L) in women or receipt of drug treatment for reduced HDL-C; (4) Systolic blood pressure ≥ 130 mmHg or diastolic blood pressure ≥ 85 mmHg or receipt of treatment of previously diagnosed hypertension; (5) Elevated plasma glucose (FPG) ≥ 5.6 mmol/dL or receipt of drug treatment for elevated glucose [7].

According to the JCDCG definition, MetS was defined as if there were three or more of the following abnormalities: (1) Central obesity (WC $\ge$ 90 cm for men and $\ge$ 85 cm for women); (2) Elevated triglyceride level $\ge$ 1.7 mmol/L) or receipt of specific treatment for this lipid abnormality; (3) Reduced HDL-C level (< 1.0 mmol/l) or specific treatment for this lipid abnormality; (4) Systolic blood pressure ≥ 130 mmHg or diastolic blood pressure ≥ 85 mmHg or current treatment for hypertension or previously diagnosed hypertension; (5) Elevated fasting plasma glucose level (FPG $\ge$ 6.1 mmol/L or 2 h postprandial PG ≥ 7.8 mmol/L) or previously diagnosed diabetes mellitus [9].

### Statistical analysis

The study population was sampled with the multilevel, stratified sampling design based on sex, area, and province [19]. Survey weights were computed based on the study design and 2010 Chinese census data and included oversampling for specific age subgroups, nonresponse, and other demographics between the sample and the total population. Differential probabilities of selection were adjusted, and the complex sampling design was used to enhance the representativeness of the survey sample population.

All data analyses were conducted using R version 4.1.1(http://www.r-project.org). The normality of the data was assessed by the Kolmogorov–Smirnov test. Means for continuous variables and percentages and proportions for categorical variables were used for summarizing. The Student t-test and Rao-Scott χ2 test were used to assess the differences across groups for continuous and categorical variables. Venn diagrams and kappa value ( poor, kappa ≤ 0.20; fair, kappa = 0.21–0.40; moderate, kappa = 0.41–0.60; substantial, kappa = 0.61–0.80; very good, kappa > 0.80) were used to assess disparity and agreement of three definitions. Univariate analysis was conducted to identify variables potentially associated with any defined MetS, and variables with *P* < 0.10 were included in the multivariable logistic regression. The 95% confidence intervals (CIs) were calculated for Odds ratios (OR). All tests were two-tailed, and a value of *P* < 0.05 was considered statistically significant.

## Result

### Characteristics of the study population

A total of 13,035(45.4%) men and 15,682(54.6%) women aged ≥ 35 years old were included in this survey. The characteristics of the participants are shown in Table 1. Overall, the mean age was 52.0 years (51.5 years for men and 52.4 years for women), and the range of age was 35 to 107 years. Most (65.8%) people lived in rural areas, and 40.6% were located in eastern China, 81.4% were educated in middle school or below, and 12.8% of participants had a CVD family history. In men, 48.3% were current smokers, and 37.9% had alcohol use, whereas the corresponding proportions were only 2.6% and 2.7% in women. Compared to women, men had a higher level of WC, TG, blood pressure, fasting plasma glucose, and lower levels of HDL-C.

**Table1:** Characteristics of the study population

	**Total**	**Men**	**Women**	*P*
	(*N* = 28,717)	(*N* = 13,035)	(*N* = 15,682)	
Age (years)	52.0(51.2–52.7)	51.5(50.8–52.3)	52.4(51.7–53.1)	0.001
Region (*n* %)				0.889
East	40.6(28.6–52.7)	40.3(27.4–53.1)	41.0(29.6–52.5)	
Central	32.0(16.5–47.6)	32.3(16.0–48.6)	31.8(16.9–46.7)	
West	27.3(17.3–37.3)	27.4(17.6–37.3)	27.2(16.9–37.5)	
Area (*n* %)				0.792
Rural	65.8(46.3–85.3)	65.6(45.2–86.0)	66.0(47.3–84.8)	
Urban	34.2(14.7–53.7)	34.4(14.0–54.8)	34.0(15.2–52.7)	
Education level (*n* %)				< 0.001
Middle school or below	81.4(75.3–86.2)	77.7(71.2–83.0)	85.1(79.3–89.6)	
High school or vocational school	14.0(10.6–18.3)	16.8(13.2–21.2)	11.2(7.9–15.5)	
College and above	4.6(3.0–7.1)	5.5(3.6–8.4)	3.7(2.3–5.9)	
Smoking status (*n* %)				< 0.001
No	74.3(72.3–76.4)	51.7(47.7–55.7)	97.4(95.7–98.4)	
Yes	25.7(23.6–27.7)	48.3(44.3–52.3)	2.6(1.6–4.3)	
Alcohol use (*n* %)				< 0.001
No	79.5(76.0–82.6)	62.1(56.0–68.3)	97.3(96.0–98.1)	
Yes	20.5(17.4–24.0)	37.9(31.7–44.0)	2.7(1.9–4.0)	
WC (cm)	83.65(82.04–85.26)	85.71(84.21–87.21)	81.55(79.79–83.31)	< 0.001
TG (mmol/L)	1.48(1.41–1.55)	1.56(1.49–1.64)	1.40(1.32–1.47)	< 0.001
HDL (mmol/L)	1.31(1.26–1.37)	1.27(1.21–1.32)	1.36(1.31–1.42)	< 0.001
FPG (mmol/L)	5.52(5.36–5.68)	5.58(5.43–5.73)	5.45(5.28–5.63)	0.008
SBP (mmHg)	131.03(129.85–132.20)	131.70(130.64–132.75)	130.35(128.83–131.86)	0.025
DBP (mmHg)	78.08(77.36–78.80)	80.16(79.28–81.04)	75.96(75.11–76.80)	< 0.001
BMI (kg/m2)	24.57(24.06–25.09)	24.57(24.11–25.04)	24.58(23.99–25.16)	0.971
Family history of CVD (n %)				< 0.001
No	87.2(82.6–90.8)	88.4(83.7–91.9)	86.0(81.4–89.6)	
Yes	12.8(9.2–17.4)	11.6(8.1–16.3)	14.0(10.4–18.6)	

Data are shown as values(95%CI)

*WC* Waist circumference, *TG* Triglycerides, *HDL* High-density lipoprotein cholesterol, *LDL* Low-density lipoprotein cholesterol, *SBP* Systolic blood pressure, *DBP* Diastolic blood pressure, *FPG* Fasting plasma glucose, *BMI* Body mass index, *CVD* Coronary cardiovascular disease

### Prevalence and presence of MetS in different definitions

Table 2 shows the prevalence of MetS with IDF, the revised ATP III, and JCDCG criteria. The prevalence of MetS in the overall population was 26.4% (22.2% in men and 30.3% in women) by IDF criteria, 32.3% (29.2% in men and 35.4% in women) by revised ATP III definition, 21.5% (24.4% in men and 18.5% in women) by JCDCG criteria. Despite some subtle differences, the relationship between various factors and MetS according to the three definitions were very similar. Regardless of the definition used, living in urban areas, having a family history of CVD, or having a higher BMI was significantly associated with a higher prevalence of MetS in the overall population and in both men and women. The prevalence of MetS reached its highest in the age group of 55–64 years in the total population and 45–54 years in men, and the prevalence decreased with age regardless of the definition used. In women over 55 years of age, the MetS prevalence maintained a high level. Regardless of the definition used, higher education levels were associated with a higher prevalence of MetS in men. In contrast, higher education levels were associated with a lower prevalence of MetS in women. The difference was statistically significant in the overall population only when the JCDCG definition was used and significant in women when IDF and the revised ATP III definitions were used. For smoking, there was a significant association between smoking and MetS in the overall population, but not in men and women. Regardless of the definition used, alcohol use was associated with a lower prevalence of MetS in women, whereas when using the IDF and the revised ATP III, alcohol use was associated with a higher prevalence of MetS in men. In contrast, in the overall population, alcohol use was only significantly associated with MetS as defined by the JCDCG.

**Table 2:** The Prevalence of MetS defined by different definitions

	IDF %			Revised ATP III%			JCDCG %		
	Total	Men	Women	Total	Men	Women	Total	Men	Women
Total	26.4	22.2	30.3	32.3	29.2	35.4	21.5	24.4	18.5
Age group									
35–44	19.5	21.6	17.3	24.4	28.2	20.4	16.4	23.8	8.2
45–54	27.7	24.3	31.3	33.4	31.4	35.5	22.0	26.3	17.5
55–64	32.7	23.0	42.3	39.4	30.4	48.4	26.6	25.5	27.8
65–74	31.4	19.5	42.8	37.9	26.9	48.4	24.9	21.2	28.5
≥ 75	30.3	17.5	40.1	38.1	24.2	48.7	24.5	19.3	28.4
*P*	< 0.0001	0.0146	< 0.0001	< 0.0001	0.0362	< 0.0001	< 0.0001	0.0320	< 0.0001
Region									
East	31.6	28.0	35.2	36.4	34.0	38.9	24.9	28.4	21.3
Central	23.0	16.7	29.6	30.3	25.4	35.4	19.6	21.1	17.9
West	22.8	20.3	25.3	28.4	26.8	30.1	18.6	22.4	14.8
*P*	0.0024	< 0.0001	0.0418	0.0058	< 0.0001	0.0511	0.0021	0.0004	0.0273
Area									
Rural	22.9	18.2	27.5	28.5	24.9	32.2	18.6	20.9	16.2
Urban	33.4	29.9	36.9	39.5	37.5	41.6	27.1	31.1	22.9
*P*	< 0.0001	< 0.0001	0.0023	< 0.0001	< 0.0001	0.0065	0.0001	< 0.0001	0.0089
Education level									
Middle school or below	26.0	20.5	31.2	31.8	27.3	35.9	20.9	23.1	18.8
High school or vocational school	28.8	27.3	31.2	34.9	34.9	34.9	24.1	28.1	18.1
College and above	26.7	31.9	18.9	33.1	38.9	24.2	24.3	32.2	12.1
*P*	0.1303	< 0.0001	0.0103	0.0814	< 0.0001	0.0095	0.0188	< 0.0001	0.1023
Smoking status									
No	27.7	22.1	30.7	33.5	30.0	35.4	20.8	25.1	18.4
Yes	22.8	22.4	30.7	28.8	28.4	35.3	23.5	23.7	19.6
*P*	0.0011	0.8684	0.9979	0.0016	0.1890	0.9753	0.0240	0.1725	0.7554
Alcohol use									
No	26.9	20.5	31.0	32.7	28.1	35.7	20.7	23.7	18.7
Yes	24.7	25.0	20.5	30.7	31.1	24.6	24.5	25.5	10.0
*P*	0.1184	< 0.0001	< 0.0001	0.1514	0.0063	< 0.0001	0.0011	0.0723	< 0.0001
BMI group									
Underweight	0.6	0.5	0.6	2.3	1.7	3.0	1.3	1.2	1.4
Normal range	6.9	2.3	11.3	13.8	9.1	18.4	6.9	6.9	6.9
Overweight	33.8	27.8	40.8	40.5	36.9	44.6	26.7	30.3	22.5
Obesity	64.8	65.9	63.8	66.4	68.4	64.6	50.8	60.5	42.2
*P*	< 0.0001	< 0.0001	< 0.0001	< 0.0001	< 0.0001	< 0.0001	< 0.0001	< 0.0001	< 0.0001
Family history of CVD (n %)									
No	24.6	20.7	28.5	30.3	27.7	33.1	20.0	23.0	16.8
Yes	39.4	33.7	44.2	45.8	41.2	49.6	31.8	35.3	28.9
*P*	< 0.0001	< 0.0001	< 0.0001	< 0.0001	< 0.0001	< 0.0001	< 0.0001	< 0.0001	< 0.0001

*MetS* Metabolic syndrome, *IDF* International Diabetes Federation; Revised ATP III: the American Heart Association/National Heart, Lung, and Blood Institute updated the ATP III; *JCDCG* The Joint Committee for Developing Chinese Guidelines, *BMI* Body mass index, *CVD* Cardiovascular disease

### Agreement on the various definitions of the metabolic syndrome

**Fig. 1:**
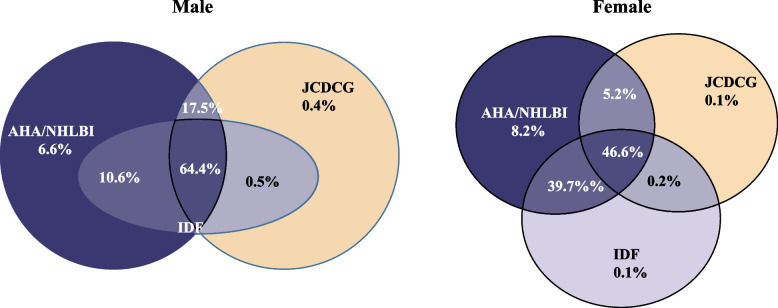
Venn diagrams showing the agreement and disparity in the diagnosis of the metabolic syndrome defined by IDF, the revised ATP III and JCDCG criteria among those 3879 men and 6288 women who qualified for the diagnosis of the metabolic syndrome by at least one of these definitions. Abbreviations: IDF: International Diabetes Federation; the revised ATP III: the American Heart Association/National Heart, Lung, and Blood Institute updated the ATP III; JCDCG: the Joint Committee for Developing Chinese Guidelines

The consistency and differences between diagnoses using IDF, the revised ATP III, and JCDCG criteria are shown in Fig. 1 and Table 3. In individuals with MetS diagnosed according to at least one definition, 64.4% of men and 46.6% of women were diagnosable by all definitions, and above 90% of people diagnosed with MetS according to two or three definitions. The JCDCG definition was the strictest, especially for women, only 52.1% of women were diagnosed with MetS. Table 3 shows the kappa values between any two definitions for men and women. The test showed good consistency between any two definitions in men and between the revised ATP III and IDF in women, with kappa values ranging from 0.77 to 0.89. JCDCG was moderately consistent with IDF (kappa = 0.58) and the revised ATP III (kappa = 0.58) in women.

**Table 3:** The agreement between the various definitions of the MetS

	Revised ATP III		JCDCG	
	kappa	95%CI	kappa	95%CI
Men				
IDF	0.81	0.75–0.87	0.77	0.73–0.80
Revised ATP III			0.86	0.85–0.88
Women				
IDF	0.89	0.86–0.92	0.58	0.56–0.60
Revised ATP III			0.58	0.56–0.60

*MetS* Metabolic syndrome, *CI* Confidence Interval, *IDF* International Diabetes Federation, Revised ATP III: the American Heart Association/National Heart, Lung, and Blood Institute updated the ATP III; *JCDCG* The Joint Committee for Developing Chinese Guidelines

### Multivariable logistic regression analysis of factors related to MetS

Table 4 shows the factors associated with MetS in men and women according to different definitions. The results showed that area, age, education, smoking, alcohol use, and family history of cardiovascular disease were related to MetS, but the effects and correlation intensity of these factors varied with gender and definition. Living in urban areas and having a family history of CVD was significantly associated with the high prevalence of MetS in both men and women under all MetS definitions, although there were slight differences in OR values. Among men, 75 years and older were significantly associated with a lower prevalence of MetS, and college education and above were significantly associated with a higher prevalence of MetS according to all three definitions. However, among women, regardless of the definition used, all groups aged 45 and above were significantly associated with a higher prevalence of MetS, and college education and above and alcohol use were significantly associated with a lower prevalence of MetS. Living in the eastern region was significantly associated with a higher prevalence of MetS in men but not in women. Smoking was significantly associated with a lower prevalence of MetS defined by the revised ATP III and JCDCG criteria but not associated with MetS by IDF in men and according to all three definitions in women. In men, alcohol use was only significantly associated with a higher prevalence of MetS defined by IDF criteria. In women, alcohol use was associated with a lower prevalence of MetS defined by all three definitions.

**Table 4:** Factors related to MetS defined by IDF, Revised ATP III, and JCDCG definitions

		IDF		AHA		JCDCG	
		Men	Women	Men	Women	Men	Women
Area	Rural	reference	reference	reference	reference	reference	reference
	Urban	1.62(1.32–1.98)^‡^	1.53(1.13–2.06)^†^	1.59(1.39–1.81)^‡^	1.51(1.16–1.97)^†^	1.53(1.35–1.74)^‡^	1.50(1.14–1.97)^†^
Age	35–44	reference		reference	reference	reference	reference
	45–54	1.06(0.92–1.21)	2.01(1.52–2.65)^‡^	1.08(0.93–1.25)	2.01(1.63–2.47)^‡^	1.07(0.90–1.26)	2.18(1.79–2.66)^‡^
	55–64	0.98(0.82–1.18)	3.19(2.34–4.35)^‡^	1.03(0.87–1.22)	3.39(2.62–4.40)^‡^	1.00(0.84–1.20)	3.92(3.26–4.71)^‡^
	65–74	0.86(0.72–1.03)	3.38(2.53–4.52)^‡^	0.91(0.81–1.03)	3.50(2.69–4.57)^‡^	0.82(0.70–0.97)^*^	4.19(3.13–5.61)^‡^
	≥ 75	0.75(0.59–0.96)^*^	3.04(2.25–4.10)^‡^	0.79(0.66–0.93)^†^	3.59(2.78–4.63)^‡^	0.72(0.58–0.89)^†^	4.23(2.97–6.03)^‡^
Education level	Middle school or below	reference	reference	reference	reference	reference	reference
	High school or vocational school	1.17(1.07–1.27)^†^	0.91(0.80–1.05)	1.17(1.02–1.35)^*^	0.90(0.77–1.05)	1.07(0.95–1.21)	0.92(0.81–1.04)
	College and above	1.41(1.13–1.77)^†^	0.57(0.41–0.78)^†^	1.35(1.13–1.61)^†^	0.65(0.49–0.87)^†^	1.27(1.05–1.52)^*^	0.72(0.54–0.95)^*^
Smoking status	No	reference	reference	reference	reference	reference	reference
	Yes	0.92(0.77–1.11)	0.76(0.57–1.01)	0.86(0.79–0.94)^†^	0.73(0.51–1.05)	0.87(0.80–0.95)^†^	0.80(0.53–1.21)
Alcohol use	No	reference	reference	reference	reference	reference	reference
	Yes	1.19(1.08–1.31)^†^	0.50(0.38–0.65)^‡^	1.10(0.97–1.26)	0.51(0.43–0.62)^‡^	1.04(0.90–1.20)	0.42(0.35–0.52)^‡^
Region	West	reference	reference	reference	reference	reference	reference
	Central	0.81(0.56–1.19)	1.31(0.75–2.26)	0.97(0.76–1.23)	1.35(0.87–2.08)	0.96(0.77–1.21)	1.33(0.86–2.05)
	East	1.35(1.02–1.8)^*^	1.45(0.86–2.43)	1.27(1.03–1.56)^*^	1.33(0.83–2.14)	1.26(1.05–1.50)^*^	1.39(0.89–2.18)
Family history of CVD	No	reference	reference	reference	reference	reference	reference
	Yes	1.65(1.48–1.84)^‡^	1.55(1.41–1.69)^‡^	1.60(1.45–1.77)^‡^	1.58(1.40–1.78)^‡^	1.62(1.45–1.81)^‡^	1.56(1.37–1.77)^‡^

^*^P < 0.05, ^†^*P* < 0.01, ^‡^*P* < 0.001; OR (95%CI), calculated with multivariable logistic regression stratified by sex

*MetS* Metabolic syndrome, *OR* Odds Ratio, *CI* Confidence Interval, *IDF* International Diabetes Federation; Revised ATP III: the American Heart Association/National Heart, Lung, and Blood Institute updated the ATP III; *JCDCG* The Joint Committee for Developing Chinese Guidelines, *BMI* Body mass index, *CVD* Cardiovascular disease. Factors in the model: age, area, education level, smoking status, alcohol use, region, and family history of CVD

## Discussion

This study aimed to investigate the prevalence and characteristics of MetS with different definitions across China. The results showed that the overall prevalence of MetS among Chinese populations aged ≥ 35 years according to the definition of IDF, the revised ATP III, and JCDCG was 26.4%, 32.3%, and 21.5%, respectively. The MetS was less prevalent in men than women according to IDF definition (22.2% vs 30.3%) and the revised ATP III (29.2% vs 35.4%) definition, but the opposite was true according to JCDCG definition (24.4%vs 18.5%). The result also showed that JCDCG definition was not in good agreement with IDF and the revised ATP III in women. In addition, the study indicated that area, age, education, smoking, alcohol use, and family history of CVD were related to MetS, but the impact and strength of the association of these factors varied by gender and definition.

The study explored the prevalence and characteristics of MetS with different MetS definitions across China. The prevalence of MetS varied greatly, with the lowest being defined by JCDCG (21.5%) and the highest being defined by ATP III (32.3%), the latter was about 1.5 times of the former. Even if estimated according to the definition of the lowest prevalence, MetS was common in the Chinese adults. Therefore, it is necessary to take targeted intervention measures to reduce the burden of MetS in China. Multivariate logistic regression showed that although the impact and correlation intensity varied by gender and definition, region, age, education, smoking, alcohol consumption, and family history of CVD were factors associated with MetS. An in-depth study of the relationship between these factors and MetS may help to understand the causes of MetS and help to control MetS.

Consistency and difference analysis showed that there was a great overlap between the three definitions. Among individuals with MetS diagnosed according to at least one definition, 64.4% of men and 46.6% of women could be diagnosed by all definitions. This may explain why the influence and correlation intensity of the factors associated with MetS varied by definition, but the difference was not large. The consistency tests showed that the consistency between any two definitions of men and the revised ATP III definition and IDF definition of women was relatively good, while the consistency between JCDCG and IDF definition (kappa 0.58) and the revised ATP III (kappa 0.58) was relatively poor in women. Moreover, the results showed that the MetS prevalence was higher in men than in women with IDF and the revised ATP III definition, but lower in men than in women with the JCDCG definition. This phenomenon may be caused by the strictest central obesity standard (WC $\ge$ 85 cm for women). Understanding the differences among the definitions may helpful to correctly analyze the differences in prevalence among different definitions. Some studies have shown that the revised ATP III definition was the best predictor of cardiovascular disease [21, 22]. The current study is a cross-sectional study, and it is impossible to compare the advantages and disadvantages of different definitions. To solve this problem, more longitudinal studies may be needed. The prevalence of MetS in our population lies well within the data previously obtained in China [13, 21, 23]. In a nationwide studies of people over 45 years old, the prevalence of MetS was 34.8%, 39.7%, and 25.6%, according to IDF, the revised ATP III, JCDCG criteria, respectively [21]. The participants in that study were older than those in our study. In a survey of people aged 18 years and older, the prevalence of MetS according to the revised ATP III definition was 24.2%, much lower than the 32.3% we obtained when using the same definition [13]. However, the prevalence of MetS in the 45–54, 55–64, and ≥ 65 years age groups in that study was 32.12%, 36.97%, 37.81%, respectively. In our study, the MetS prevalence in the 45–54, 55–64, 65–64, and ≥ 75 years age groups was 33.4%, 39.4%, 37.9%, and 38.1%, respectively (Table 2). The numbers are very close.

It has been seen that the prevalence of MetS was closely related to age and gender [24]. In our study, the prevalence of MetS in the total population peaked at the age of 55–64 years, which is close to Wu’s study, which peaked at the age of 60–69 years [25]. In addition to age, gender cannot be ignored. In our study, the prevalence of MetS in women over 45 years old remained at a high level (Table 2), and the odds ratio of women over 45 years old reached around 3 (Table 4). Menopause may explain this phenomenon, for menopause generally occurs around the age of 50 [26]. The loss of heart and kidney protection of female hormones with age may lead to the sharp increase in hypertension and cardiovascular disease in postmenopausal women [27]. The prevalence of MetS in our study reached its highest in men aged 45–54 years and then decreased, becoming a protective factor over 65 years. This marked reversal of gender difference in older adults may be partly attributable to the men prone to metabolic disease who had died before the age of 75 or refused to participate in this study [27, 28]. The characteristics of MetS vary by sex, suggesting that reasonable comparisons should be made by sex.

In addition to age and gender, our study showed there were some other factors associated with MetS. The present study revealed that individuals living in urban areas had a higher risk of MetS, in line with some other studies [25, 29]. The reason for this phenomenon may be that, in China, compared to rural areas, in economically developed urban areas with rapid industrialization, animal food and fast food with high fat and purine content increased dramatically, while grain consumption was the opposite [30]. Our results also indicated that there were gender differences in the association between education and MetS, with a positive association for women and negative for men. This was consistent with a study conducted by the Korea National Health and Nutrition Examination Surveys [31]. One possible explanation was that more educated women might have a favorable opportunity to get more nutrition knowledge and prefer healthy food consumption patterns [32]. And men with higher education are more likely to consume high-calorie foods and alcohol, while avoiding physically demanding tasks [31]. It is worth noting that a family history of CVD was an independent risk factor for MetS in our study, suggesting that more attention should be paid to individuals with a CVD family history [33]. There was a significant negative correlation between smoking and MetS defined by the revised ATP III and JCDCG definition in men. Although the association between smoking and MetS was not significant in women, its OR value was smaller than that in men, which may be due to the small number of women smoking and insufficient test power. This phenomenon is contrary to the general conclusion that smokers had higher insulin resistance and a higher risk of fatal coronary artery disease than non-smokers [34]. One possible explanation is that some smokers weigh less than non-smokers because of the effects of nicotine on metabolism [35]. Interestingly, we found an arguable result that alcohol use was a protective factor for women and a risk factor for men, which was also reported in Sampson’s study [36]. Men have higher drinking rates and tend to consume large amount of alcohol. Heavy drinking, especially > 30 g/day in men, is often accompanied by an increase in energy intake and changes in the concentration of steroid hormones that may cause central fat storage, which will aggravate elevated blood pressure, elevated plasma glucose, and central obesity [37]. Women drink less often and in lower amounts. Some studies have shown that drinking small amounts of alcohol may have cardiovascular protective effects [38]. However, the protective effect of drinking small amounts of alcohol remains controversial and needs further study [39].

Our study has some strength. Firstly, the sample size of the current study was large and the study population was randomly selected from the whole country by stratified and multistage sampling, and the sample was nationally representative. This allowed us to estimate the prevalence of MetS across the country and to explore the impact of different definitions on the prevalence of MetS in China. Secondly, strict quality control ensured the high quality of data and reliability of the findings. The uniform research protocol and measuring instruments, strict training and examination, and the centralized detection of blood glucose and lipids in the central laboratory ensure the accuracy and comparability of the data. Thirdly, we used different definitions in the same group of people to explore the prevalence and characteristics of MetS, which enables us to have a comprehensive understanding of the prevalence and characteristics of MetS and is also convenient to compare with the data of other regions and population. The limitations of this study need to be recognized. Firstly, we only compared the revised ATP III, IDF, and JCDCG definitions due to the lack of some indicators, such as the data of insulin resistance. Secondly, we explored some related factors of MetS, but we cannot claim causality because of the cross-sectional design. Thirdly, in this study, we explored the related factors of MetS, some variables which may affect MetS were not included in our study, such as physical activity and dietary patterns. In addition, due to funding and other reasons, the investigation lasted for a long time, and some related factors may have changed.

## Conclusions

In summary, the prevalence and characteristics of metabolic syndrome vary according to the definition used in the Chinese population. The three MetS definitions of IDF, the revised ATP III, and JCDCG are in relatively good agreement in men, but the differences between JCDCG and IDF and between JCDCG and the revised ATP III are large in women. On the other hand, even if estimated according to the definition of the lowest prevalence. MetS is common in the Chinese adults. It is necessary to explore the causes of the difference in the prevalence of MetS in different populations and take targeted intervention measures in China.

## Supplementary Information

**Additional file 1: Appendix Table 1.** Characteristics of the subjects included and excluded in the analysis (age ≥ 35 years old).

**Additional file 2: Supplemental Text 2.** Weights calculation in the Study.

## Data Availability

The dataset analyzed during the current study is available from the corresponding author on reasonable request.

## Abbreviations

MetS: Metabolic syndrome
WHO: The World Health Organization
EGIR: The European Group for the study of Insulin Resistance
NCEP: The National Cholesterol Education Program Adult Treatment Panel III
IDF: The International Diabetes Federation
Revised ATP III: The American Heart Association/National Heart, Lung, and Blood Institute updated the ATP III
JCDCG: The Joint Committee for Developing Chinese Guideline
WC: Waist circumference
TG: Triglyceride
HDL-C: High-density lipoproteins cholesterol
BP: Blood pressure
FPG: Fasting plasma glucose
