# Supplementary material for: The prevalence and characteristics of metabolic syndrome according to different definitions in China: a nationwide cross-sectional study, 2012–2015
Source: BMC Public Health. 2022 Oct 7;22:1869. doi: 10.1186/s12889-022-14263-w (PMC9540728; doi:10.1186/s12889-022-14263-w)
Supplement: Supplementary file 1 — Additional file 1: Appendix Table 1. Characteristics of the subjects included and excluded in the analysis (age ≥ 35 years old). [file 12889_2022_14263_MOESM1_ESM.docx]

Appendix Table 1. Characteristics of the subjects included and excluded in the analysis (age ≥ 35 years old)

|  | **Included(N=28717)** | |  | **Excluded (N=6277)** | | *p* value |
| --- | --- | --- | --- | --- | --- | --- |
|  | n | statistics |  | n | Statistics |  |
| Age (years) | 28717 | 52.0(51.2-52.7) |  | 6277 | 51.7(49.6-53.9) | <0.001 |
| region (n %) |  |  |  |  |  | <0.001 |
| East | 12119 | 40.6(28.6-52.7) |  | 3119 | 46.41(34.67-58.16) |  |
| Central | 11040 | 32.0(16.5-47.6) |  | 2257 | 29.93(20.32-39.54) |  |
| West | 5558 | 27.3(17.3-37.3) |  | 901 | 23.65(13.25-34.06) |  |
| Area (n %) |  |  |  |  |  | <0.001 |
| urban | 15376 | 65.8(46.3-85.3) |  | 2536 | 58.58(40.59-76.58) |  |
| rural | 13341 | 34.2(14.7-53.7) |  | 3741 | 41.42(23.42-59.41) |  |
| Education level (n %) | | |  |  |  | <0.001 |
| Middle school or below | 22796 | 81.4(75.3-86.2) |  | 4856 | 80.49(74.99-85.98) |  |
| High school or vocational school | 4064 | 14.0(10.6-18.3) |  | 855 | 13.74(9.15-18.32) |  |
| College and above | 1857 | 4.6(3.0-7.1) |  | 496 | 5.77(3.37-8.18) |  |
| Smoking status (n %) |  |  |  |  |  | 0.008 |
| No | 22378 | 74.3(72.3-76.4) |  | 4770 | 76.37(72.92-79.82) |  |
| Yes | 6339 | 25.7(23.6-27.7) |  | 1231 | 23.63(20.18-27.08) |  |
| Alcohol use (n %) |  |  |  |  |  | 0.145 |
| No | 23308 | 79.5(76.0-82.6) |  | 4913 | 79.39(76.56-82.22) |  |
| Yes | 5409 | 20.5(17.4-24.0) |  | 1202 | 20.61(17.78-23.44) |  |
| WC (cm) | 28717 | 83.65(82.04-85.26) |  | 5773 | 84.58(82.58-86.59) | <0.001 |
| TG (mmol/L) | 28717 | 1.48(1.41-1.55) |  | 1445 | 1.37(1.25-1.49) | 0.299 |
| HDL (mmol/L) | 28717 | 1.31(1.26-1.37) |  | 3230 | 1.30(1.23-1.37) | <0.001 |
| FPG (mmol/L) | 28717 | 5.52(5.36-5.68) |  | 3019 | 5.22(4.93-5.50) | <0.001 |
| SBP (mmHg) | 28717 | 131.03(129.85-132.20) |  | 5864 | 132.78(128.91-136.64) | <0.001 |
| DBP (mmHg) | 28717 | 78.08(77.36-78.80) |  | 5826 | 79.46(77.09-81.82) | <0.001 |
| BMI (kg/m2) | 28717 | 24.57(24.06-25.09) |  | 5717 | 24.84(24.08-25.60) | <0.001 |
| CVD family history (n %) | | |  |  |  | 0.003 |
| No | 24285 | 87.2(82.6-90.8) |  | 5403 | 88.98(86.31-91.64) |  |
| Yes | 4432 | 12.8(9.2-17.4) |  | 874 | 11.02(8.36-13.69) |  |

Data shown as values(95%CI). All values were weighted to represent the total population of Chinese aged 18 years or older based on Chinese census 2010.

Abbreviations: WC, waist circumference; TC, total cholesterol; TG, triglycerides; HDL, high-density lipoprotein cholesterol; LDL, low-density lipoprotein cholesterol; SBP, Systolic blood pressure; DBP, Diastolic blood pressure; FPG, fasting plasma glucose; BMI, body mass index; CVD, coronary cardiovascular disease.
